# Therapeutic Writing in a Group Setting for Girls with ADHD: A Case Series on Self-Perception, Self-Esteem, and Socio-Emotional Functioning

**DOI:** 10.3390/healthcare14162566

**Published:** 2026-08-17

**Authors:** Adriana Piccolo, Marcella Di Cara, Silvia Messina, Carmela De Domenico, Alessia Fulgenzi, Daniele Borzelli, Caterina Impallomeni, Emanuela Tripodi, Angelo Quartarone, Francesca Cucinotta

**Affiliations:** 1 IRCCS Centro Neurolesi “Bonino Pulejo”, 98124 Messina, Italy; adriana.piccolo@irccsme.it (A.P.); marcella.dicara@irccsme.it (M.D.C.); messinasilvia67@gmail.com (S.M.); carmela.dedomenico@irccsme.it (C.D.D.); alessia.fulgenzi@irccsme.it (A.F.); caterina.impallomeni@irccsme.it (C.I.); emanuela.tripodi@irccsme.it (E.T.); angelo.quartarone@irccsme.it (A.Q.); 2 Laboratory of Physiology, Department of Translational Medicine, Università del Piemonte Orientale, 28100 Novara, Italy; daniele.borzelli@uniupo.it

**Keywords:** ADHD, therapeutic writing, adolescents, female, self-esteem, case series, rehabilitation

## Abstract

Background: Attention-deficit/hyperactivity disorder (ADHD) is frequently associated with emotional dysregulation, socio-emotional difficulties, and low self-esteem. Narrative-based approaches, including therapeutic writing, may support identity development and promote adaptive self-representations. However, there is limited evidence regarding structured, group-based therapeutic writing interventions for this population, particularly for female subjects. This case series provides preliminary and exploratory evidence regarding the feasibility of and potential changes associated with this approach in female adolescents with ADHD. Methods: This case series included four adolescents (aged 11–14 years) with a clinical diagnosis of ADHD and presenting with emotional and socio-emotional difficulties. Participants underwent a structured group intervention consisting of seven sessions integrating therapeutic writing techniques and cognitive–behavioral principles, supervised by a specialized psychotherapist. The following outcome measures were collected at baseline (t0) and post-intervention (t1): Difficulties in Emotion Regulation Scale (DERS-SF), Difficulties in Emotion Regulation Scale (SSIS-SEL), and Rosenberg Self-Esteem Scale (RSE_TOT). Data were analyzed using linear mixed-effects models, with results interpreted as exploratory estimates of pre–post changes. Results: Following the intervention, positive pre–post changes were observed in socio-emotional competencies and self-esteem. Statistically significant pre–post changes were observed in self-esteem and in socio-emotional competencies, particularly in self-awareness and self-regulation. Reductions were observed in emotional regulation difficulties, although these changes did not reach statistical significance. Conclusions: This case series suggests that a structured group-based therapeutic writing intervention may be associated with improvements in self-esteem and selected aspects of socio-emotional functioning in adolescents with ADHD. Given the small sample size and exploratory design, findings should be interpreted cautiously and require confirmation in larger controlled studies. These findings contribute preliminary evidence to an underexplored area in the underrepresented female ADHD population and highlight the potential value of integrating narrative processes within group interventions to support both individual meaning-making and interpersonal validation.

## 1. Introduction

Attention-deficit/hyperactivity disorder (ADHD) is one of the most common neurodevelopmental conditions in childhood and adolescence [1]. ADHD is characterized by persistent patterns of inattention, hyperactivity, and impulsivity that may affect behavioral regulation [2]. These traits may interfere with daily activities and with the demands of structured environments, such as schools, leading to difficulties in adaptation and experiences of frustration [3,4]. These difficulties frequently emerge in contexts that require high levels of organization, behavioral regulation, and sustained attention, skills that may be particularly challenging for many young people with ADHD [5]. Specifically, individuals with ADHD are more likely to experience episodes of exclusion or bullying in social situations, which can lead to a strong sense of stigma and negatively affect their quality of life over time [6,7]. These experiences may contribute to the formation of a self-representation primarily centered on difficulties during adolescence, a crucial stage for the development of personal and social identity, thereby influencing both self-esteem and a sense of self-efficacy [8]. The relevance of self-perception is particularly important during adolescence, a developmental period characterized by increasing vulnerability to mental health difficulties, including a growing burden of depressive disorders among adolescents and young adults [9]. Moreover, the internalization of stigmatizing experiences may negatively affect self-esteem and contribute to social withdrawal, further highlighting the importance of self-perception during this developmental stage [10]. Females with ADHD, particularly, show poorer coping skills and lower self-esteem than their male peers [11]. Furthermore, females typically experience more internalizing problems, such as anxiety and mood disorders, and frequently report self-harm and suicidal thoughts [11,12,13,14]. Compared to males with ADHD, females seem to be less aware of their own dysfunctional behavior and are often victims of bullying [15]. Finally, in terms of social functioning, girls with ADHD tend to exhibit greater verbal aggression and a higher tendency towards relational aggression than girls without ADHD, resulting in difficulties in social relationships [11]. This population appears to be underrepresented in clinical research, and there are few specific treatments based on gender differences in ADHD [16].

In recent years, several studies have begun to shift attention toward the positive aspects of this condition [17]. Indeed, some studies suggest that individuals with ADHD tend to possess a more flexible network of mental associations [18]. Typical characteristics, such as increased impulsivity and a certain degree of distractibility, have been associated with better performance in tasks requiring creativity and divergent thinking [19,20]. These findings suggest that some cognitive processing patterns commonly observed in ADHD may facilitate the generation of original ideas and the exploration of unconventional solutions. However, despite the growing body of scientific evidence highlighting these potential strengths, awareness of such resources is not always present among adolescents with ADHD. Many young people continue to develop a self-perception that is primarily centered on behavioral difficulties and academic failures, rather than a balanced self-perception based on strengths and weaknesses similar to that of their peers [7,21]. This deficit-based profile is often reinforced by educational and social contexts that tend to focus more on correcting problematic behaviors than on recognizing and fostering individual strengths [22,23].

Furthermore, health and social care systems often focus on curing or “normalizing” the typical behaviors of ADHD, rather than on supporting people and implementing compensatory strategies—both personal and environmental. A global social environment where negative opinions about ADHD and people with ADHD are frequently expressed—as has been demonstrated for other neurodevelopmental conditions, such as autism [24]—can contribute not only to masking behaviors but also to a deterioration in an individual’s self-perception and perceived quality of life [25].

However, some studies have shown different priorities among the individuals involved [26], highlighting an urgent need to improve neurodiversity-affirmative support [27]. In a therapeutic context, the term “neuro-affirming” refers to a supportive, strengths-based approach that does not rely solely on diagnosis, nor does it aim to normalize behavior or fill a gap [28]. Adopting a neurodiversity-affirmative approach to intervention appears to encourage self-acceptance and enhance coping mechanisms. Support measures and environmental accommodations that reflect individuals’ preferences are necessary to provide individuals and families who embrace neurodiversity with the necessary resources.

For this reason, interventions that promote a shift in perspective, helping young people to recognize and consciously use their positive characteristics, are increasingly considered important and represent an innovative direction in the support and management of this condition [29]. Accordingly, interventions that allow adolescents to actively reprocess their experiences and construct new self-narratives may represent a promising strategy for promoting a more balanced and integrated self-concept [30].

To address this aim, approaches based on narrative practices and writing may provide a valuable space for exploring and reprocessing personal experiences [30]. Narrative represents a fundamental tool through which individuals organize their experiences, assign meaning to life events, and construct their identity [31,32]. Through self-narration, individuals can question limiting narratives and develop new perspectives on their abilities and personal resources [33].

More recently, narrative-based approaches, including story mapping and other forms of structured narrative interventions, have been explored in adolescents with ADHD, showing potential benefits in terms of narrative organization and processing of personal experiences [16,20,34]. However, these approaches primarily focus on narrative comprehension or structured storytelling tasks, rather than on therapeutic writing as an active process of emotionally processing one’s experiences and reworking one’s self-concept.

To date, the application of therapeutic writing, conceptualized as a structured intervention aimed at facilitating the reprocessing of subjective experiences and the development of more adaptive self-representations, remains largely unexplored in adolescents with ADHD. To the best of our knowledge, no studies have examined therapeutic writing within a structured group intervention designed to promote changes in self-perception based on a neurodiversity-affirmative approach in the female ADHD population.

The primary aim of this study was to explore whether this therapeutic intervention could foster changes in self-perception, self-esteem, emotional regulation, and socio-emotional competencies.

## 2. Methods

### 2.1. Study Design

This study was designed as an A–B sequential case-series design aimed at exploring the effects of a therapeutic writing intervention on emotional and socio-relational functioning in adolescents with ADHD. This study was approved by the Local Ethics Committee, and the intervention was reported in accordance with TIDieR (Template for Intervention Description and Replication) [35] to ensure transparent, complete, and high-quality clinical case reporting. The case-series design allowed for an in-depth observation of individual and group-level changes over time within a real-world clinical setting. Given the descriptive and exploratory nature of a case-series design, no control group, randomization, or blinding procedures were implemented. All procedures were conducted following a standardized protocol to ensure consistency across sessions. No changes to the study design or assessment procedures were made after the commencement of the study.

After obtaining informed consent from legal guardians and all subjects involved in the study, all participants took part in a structured creative writing intervention delivered in a small group setting.

Outcome measures were collected at two points: before the beginning of the intervention (t0) and immediately after its completion (t1). The assessment protocol included standardized self-report measures aimed at evaluating emotional regulation, socio-emotional competencies, and self-esteem.

### 2.2. Participants and Inclusion Criteria

The study population consisted of adolescents aged between 11 and 14 years with a clinical diagnosis of ADHD who were referred to the Child Neuropsychiatry outpatient service of the IRCCS Centro Neurolesi “Bonino Pulejo” in Messina, Italy.

Inclusion criteria were: (1) a diagnosis of ADHD according to DSM-5 criteria; (2) age between 11 and 18 years; (3) female sex; (4) an intelligence quotient (IQ) ≥ 80; and (5) provision of written informed consent by parents or legal guardians, along with participant assent. Exclusion criteria included: (1) comorbidity with severe neuropsychiatric disorders (e.g., autism spectrum disorder and schizophrenia); (2) IQ ≤ 79; (3) current pharmacological treatment; and (4) absence of informed consent. Participants receiving pharmacological treatment were excluded to reduce the potential influence of medication-related effects on pre–post outcome changes. Four participants who met the inclusion criteria were enrolled in the study. Three participants had a predominantly inattentive ADHD presentation, while one participant had a combined presentation. No participants had documented psychiatric or neurodevelopmental comorbidities, and all had previously received cognitive–behavioral therapy. These clinical characteristics are summarized in Table 1.

### 2.3. Measures

Outcome measures were collected at two points: before the start of the intervention (t0) and immediately after its completion (t1). All assessments were administered using standardized self-report instruments commonly employed in clinical and research settings to evaluate emotional and psychosocial functioning. Assessments were administered by expert clinicians external to the intervention team who were not involved in the delivery of the therapeutic writing program in order to reduce potential assessor-related bias in outcome collection.

As part of the initial clinical assessment and screening procedure, participants underwent a cognitive evaluation using the Wechsler Intelligence Scale for Children—Fourth Edition (WISC-IV) [36] in order to verify eligibility criteria and ensure adequate cognitive functioning (IQ ≥ 80).

The following outcome domains were assessed:

- Emotional regulation, measured using the Difficulties in Emotion Regulation Scale (DERS-SF) [37]. The DERS-SF is a multidimensional self-report questionnaire assessing difficulties in emotional regulation across several domains, including emotional awareness, clarity, impulse control, and access to effective regulation strategies. Total scores typically range from 18 to 90, with higher scores indicating greater difficulties in emotion regulation.
- Socio-emotional competencies, assessed through the Italian version of the Social Skills Improvement System—Social Emotional Learning Edition (SSIS-SEL) [38,39]. The Italian version evaluates four core socio-emotional domains: self-awareness, self-management, social awareness, and responsible decision-making. Raw scores were used, where higher scores indicated greater socio-emotional skills and more adaptive functioning across interpersonal and emotional domains.
- Self-esteem, measured using the Rosenberg Self-Esteem Scale (RSES) [40], a widely used 10-item self-report measure of global self-worth. Total scores range from 0 to 30, with higher scores indicating higher self-esteem. Scores below 15 are generally considered indicative of low self-esteem, while scores in the mid-to-high range reflect a more positive self-evaluation.

These measures were selected to capture core domains targeted by the intervention, namely, emotional regulation, interpersonal functioning, and self-perception.

In addition, adherence to the intervention was monitored by recording participants’ attendance across the seven sessions.

### 2.4. Intervention

The therapeutic writing intervention was structured and conducted in a small group. Seven sessions of approximately two hours each were held over seven weeks. The group was led by an experienced clinical psychologist, a pediatric neuropsychiatrist, and a qualified writer, under the supervision of a specialized psychotherapist, and took place at the IRCCS Centro Neurolesi “Bonino Pulejo” in Messina, Italy.

The intervention combined the principles of narrative therapy, expressive writing [41,42], cognitive–behavioral approaches, and social learning models. The main objective was to promote emotional regulation, self-awareness, and the construction of adaptive personal narratives in adolescents with ADHD, while identifying their strengths and weaknesses.

Each session included (1) experiential activation, (2) guided reflection, (3) structured writing, and (4) group sharing. This sequence was designed to facilitate emotional engagement, cognitive processing, and the social integration of experiences. Intervention materials included colored cards, poster boards, balls, hoops, and drawing materials used to support the writing and group activities. The intervention followed the same predefined structure for all participants and was not individually tailored.

#### 2.4.1. Session 1—Group Formation and Normalization

The first session aimed to create a safe and cohesive environment to foster greater emotional sharing. Group activities and shared storytelling exercises were included to promote cohesion and reduce stigma. ADHD characteristics were redefined as shared experiences rather than deficit symptoms, fostering a new perspective on daily challenges.

#### 2.4.2. Session 2—Awareness of Attentional Processes

This session targeted metacognitive awareness of attentional functioning. Participants were guided to identify both strengths and challenges associated with their attentional style. Techniques derived from cognitive–behavioral therapy were used to facilitate self-monitoring and recognition of internal states. The use of metaphor and personalized descriptors supported cognitive defusion and increased psychological flexibility. The writing task enabled narrative processing of attentional experiences.

#### 2.4.3. Session 3—Impulsivity and Hyperactivity

The third session focused on the experiential exploration of impulsivity and hyperactivity through the use of multimodal techniques (visual stimuli, role-playing, dramatizations, etc.). The goal was to promote greater bodily awareness of behavioral patterns. In a structured and safe environment, the activities allowed participants to observe, enact, and reflect on all impulsive and hyperactive behaviors that could manifest in their daily lives. Group interaction facilitated the development of diverse perspectives. The session concluded with an autobiographical writing assignment that fostered emotional processing and the construction of coherent narratives.

#### 2.4.4. Session 4—Identification of Personal Strengths

The fourth session focused on identifying and strengthening personal resources, building on each girl’s strengths. With the guidance of experts, participants were encouraged to reflect on the personal characteristics they considered positive and the situations in which these characteristics were perceived as strengths using a solution-oriented perspective. The final group discussion and subsequent writing assignment fostered a sense of social belonging and the internalization of a more positive self-image.

#### 2.4.5. Session 5—Narrative Identity and Character Construction

This session focused on the girls’ creation of fictional characters that could represent aspects of their internal experiences. This task falls within the principles of narrative therapy, which involves the projection of internal states onto symbolic figures. Participants defined physical attributes, abilities, emotional traits, and hidden aspects of their characters, fostering identity exploration and narrative organization.

#### 2.4.6. Session 6—Problem-Solving and Executive Functioning

The sixth session focused on narrative development and problem-solving. Participants were asked to construct a problematic situation involving their character and to identify possible strategies for resolution. To support this process, a structured activity inspired by the “Tower of London” paradigm was implemented in a human, experiential format, using the metaphor of retrieving a balloon from a tree to represent goal-directed planning. This activity aimed to enhance executive functioning skills, including planning, sequencing, and cognitive flexibility, while simultaneously supporting narrative coherence and problem-solving within the story context.

#### 2.4.7. Session 7—Collective Narrative and Symbolic Integration

The final session was dedicated to the creation of a collective narrative in which individual characters interacted within a shared story. The session was structured as a symbolic journey involving conflict, emotional expression, and resolution. The narrative construction was conducted through a semi-guided storytelling process, in which therapists actively facilitated the development of the plot by providing structured prompts and guiding questions. Participants were supported through targeted inputs aimed at eliciting specific narrative elements (e.g., origin of the scenario, emergence of conflict, emotional reactions, and resolution processes). These prompts encouraged reflection on internal states, interpersonal dynamics, and coping strategies. Participants collaboratively constructed the story by responding to these guided inputs, engaging in role-based tasks, and navigating metaphorical challenges designed to reflect internal emotional processes. Therapists supported the coherence and continuity of the narrative, facilitating integration between individual contributions and promoting perspective-taking and shared meaning-making.

The intervention concluded with a reflective phase focused on personal change, the integration of the experience, and the construction of a coherent narrative meaning.

### 2.5. Statistical Analysis

Data were analyzed using built-in functions in MATLAB R2023b (MathWorks Inc., Natick, MA, USA). Owing to the repeated-measures structure of the data (baseline and post-intervention assessment for each participant), treatment effects were evaluated using separate linear mixed-effects models fitted with the fitlme function in MATLAB.

For each outcome measure, the following model was estimated:

*measure*~*isT*1 + (1|*patientId*) (1)

Here, measure represents each score derived from the DERS-SF, SSIS-SEL, and RSE_TOT questionnaires. *isT*1 is a binary variable indicating the time point of acquisition (0 = baseline, 1 = post-intervention), and *patientId* denotes the participant-specific random intercept. The fixed-effect coefficient associated with isT1 (β) represents the estimated average change from baseline to post-intervention, with positive values indicating higher scores at t1 and negative values indicating lower scores at t1 compared with baseline.

Although the study included only four participants, the mixed-effects framework was retained because it accounts for the dependency between repeated observations while providing direct estimates of the average pre–post effect. With only two repeated measurements per participant, this approach is statistically comparable to a paired pre–post comparison; however, the mixed-effects framework was retained because it provides a direct estimation of the intervention-related change while accounting for participant-level variability. Given the small sample size, the analyses were considered exploratory and primarily intended to estimate the magnitude and direction of change rather than to provide confirmatory statistical evidence.

For each model, the regression coefficient (β), standard error (SE), 95% confidence interval (CI), degrees of freedom (df), t statistic, and *p*-value were reported. In addition, Cohen’s dz was calculated as a standardized measure of the pre–post effect size. Degrees of freedom were computed according to the residual degrees-of-freedom method implemented by MATLAB’s fitlme. No adjustment for multiple comparisons was applied due to the exploratory nature of this preliminary study. A nominal threshold of *p* < 0.05 was used for statistical interpretation; however, given the exploratory design and small sample size, results were interpreted together with effect sizes and confidence intervals.

## 3. Results

The sample consisted of four female participants aged between 11 and 14 years (13.0 ± 1.41 years). All participants presented with intellectual functioning within the normal range (IQ range: 98–103), confirming the absence of cognitive impairment and supporting their ability to engage in the proposed intervention (Table 1). Adherence was 100%, with all participants attending all seven sessions.

### 3.1. Clinical Profiles and Individual Changes

#### 3.1.1. Participant 1

C. was a 14-year-old adolescent with ADHD with a predominantly inattentive presentation. At baseline, she presented difficulties in emotional regulation, frustration tolerance, and interpersonal functioning. Clinically, she appeared emotionally reactive and tended to alternate between active attempts to engage with others and relational withdrawal, particularly in situations perceived as emotionally demanding or potentially judgmental. She also showed difficulties in maintaining attention and organization during structured activities.

During the initial group sessions, C. demonstrated curiosity and interest toward the proposed writing activities, although her participation was often inconsistent. Moments of active involvement alternated with oppositional attitudes, irritability, and disengagement from the task, especially when emotional themes emerged. Her narratives initially appeared focused on conflicting experiences and themes of misunderstanding and emotional intensity.

As the intervention progressed, C. gradually became more engaged in both the writing process and group interactions. The relational climate appeared to facilitate greater emotional openness and participation, allowing her to share personal reflections more spontaneously and tolerate interpersonal exchanges more effectively. Over time, oppositional behaviors decreased, while cooperative participation and emotional sharing increased. In particular, collaborative storytelling activities seemed to foster greater perspective-taking and peer connection.

**Figure 1:**
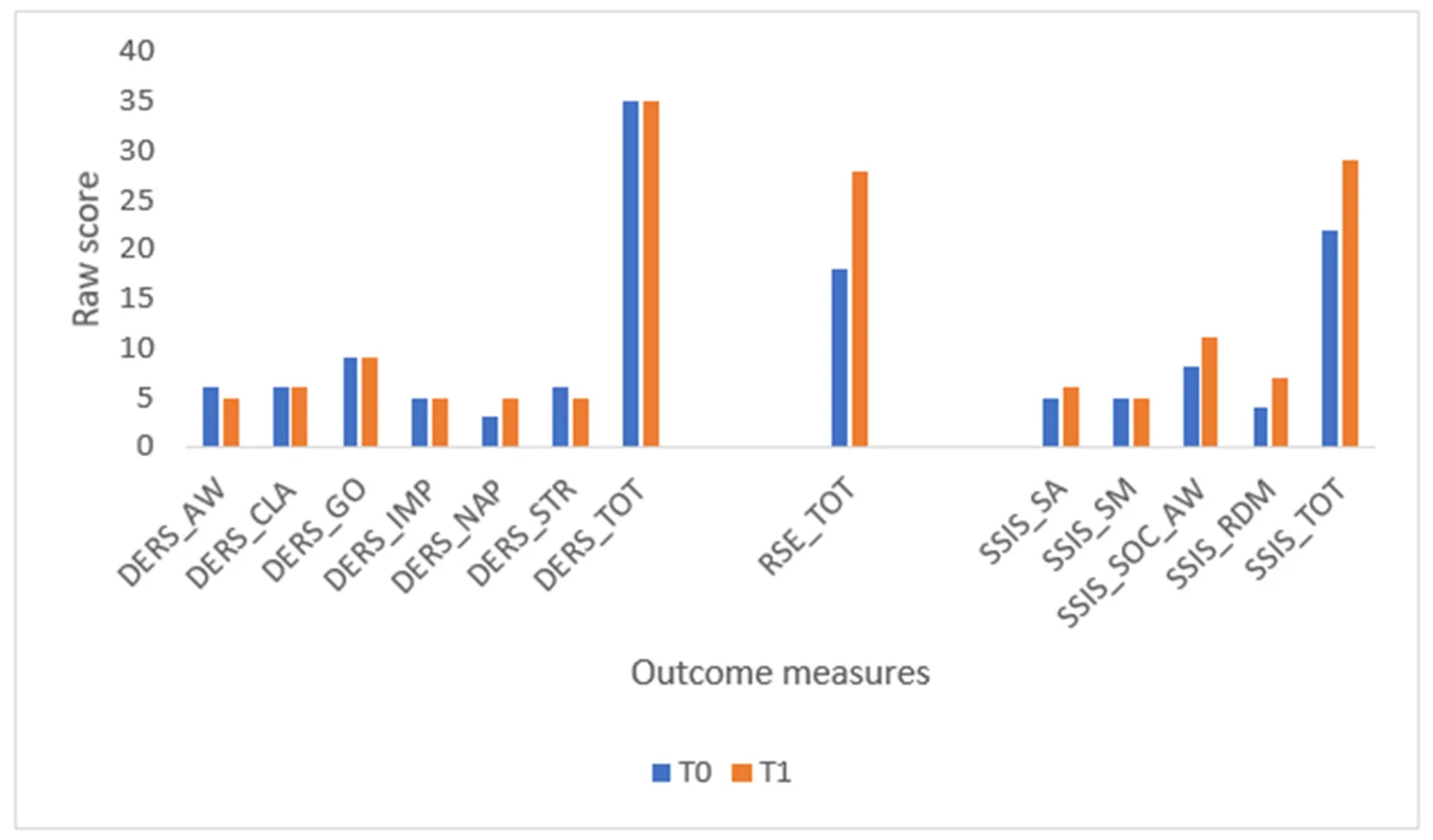
Pre- and post-intervention profile of Participant 1. Legend: DERS-SF = Difficulties in Emotion Regulation Scale; AW = awareness; CLA = clarity; GO = goals; IMP = impulse; NAP = non-acceptance; STR = strategies; TOT = total score. RSE_TOT = Rosenberg Self-Esteem Scale. SSIS-SEL = Social Skills Improvement System—Social Emotional Learning Edition; SA = self-awareness; SM = self-management; SOC_AW = social awareness; RDM = responsible decision-making.

Quantitative findings reflected partial changes in emotional functioning. Total DERS-SF scores remained stable over time (Figure 1), although slight improvements were observed in emotional awareness. Conversely, more evident changes emerged in socio-emotional functioning and self-awareness (Figure 1). A marked increase was also observed in self-esteem, as measured by RSE_TOT (Figure 1), suggesting the development of a more positive and less negatively polarized self-image following the intervention.

#### 3.1.2. Participant 2

A. was a 13-year-old adolescent diagnosed with ADHD who presented with difficulties in sustained attention, executive functioning, impulsivity, and emotional regulation. Clinically, she showed marked emotional reactivity, particularly in situations involving frustration or academic pressure, often associated with irritability. She also reported performance-related anxiety, characterized by anticipatory tension and sleep disturbances before school evaluations. Despite these difficulties, she appeared socially engaged and interested in maintaining positive peer relationships.

From the beginning of the intervention, A. showed consistent engagement and collaborative participation in group activities. She appeared attentive to her peers’ contributions and progressively demonstrated increasing openness during narrative and reflective exercises. Throughout the sessions, she showed good insight into her own difficulties and was able to identify personal resources and coping strategies within both individual writing activities and group discussions. Her cooperative attitude positively contributed to the emotional climate of the group.

**Figure 2:**
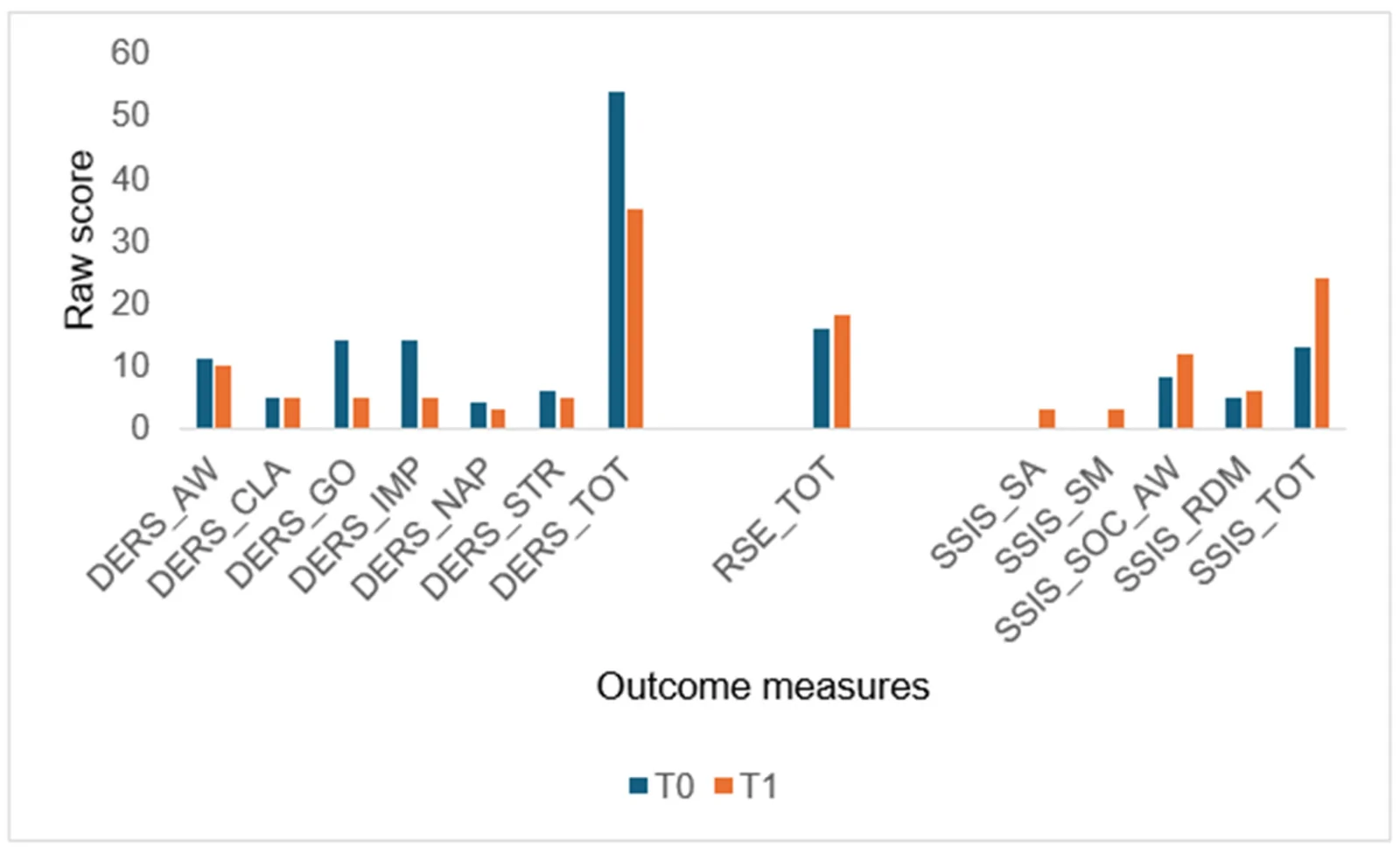
Pre- and post-intervention profile of Participant 2. Legend: DERS-SF = Difficulties in Emotion Regulation Scale; AW = awareness; CLA = clarity; GO = goals; IMP = impulse; NAP = non-acceptance; STR = strategies; TOT = total score. RSE_TOT = Rosenberg Self-Esteem Scale. SSIS-SEL = Social Skills Improvement System—Social Emotional Learning Edition; SA = self-awareness; SM = self-management; SOC_AW = social awareness; RDM = responsible decision-making.

Quantitative findings reflected significant improvements in emotional regulation, suggesting improved modulation of emotional activation and behavioral organization. More modest improvements emerged in emotional non-acceptance and emotional awareness and regulation strategies, while clarity remained stable (see Figure 2).

Socio-emotional functioning also improved. The most notable changes involved self-awareness, self-management, and social awareness, whereas responsible decision-making showed a smaller increase (see Figure 2).

Self-esteem scores on the RSE_TOT increased, indicating a modest but positive improvement in self-perception following the intervention (see Figure 2).

#### 3.1.3. Participant 3

B. was a 13-year-old adolescent diagnosed with ADHD with a predominantly inattentive presentation, associated with mild executive functioning difficulties. Clinically, she presented with distractibility, reduced persistence during cognitively demanding tasks, and difficulties in planning and organization, which negatively affected academic functioning.

During the initial phases of the intervention, B. appeared emotionally guarded and minimally involved in group interactions. Her participation was characterized by limited spontaneous sharing and reduced openness during reflective activities, often requiring external prompting to engage in discussions. However, as the sessions progressed, the supportive group environment appeared to facilitate greater participation and emotional involvement.

Over time, B. became progressively more collaborative and engaged in both narrative and relational activities. She increasingly demonstrated the ability to recognize personal strengths and resources, particularly during exercises focused on self-description and shared storytelling. Improvements were also observed in emotional expression, problem-solving attitudes, and active participation within group dynamics.

**Figure 3:**
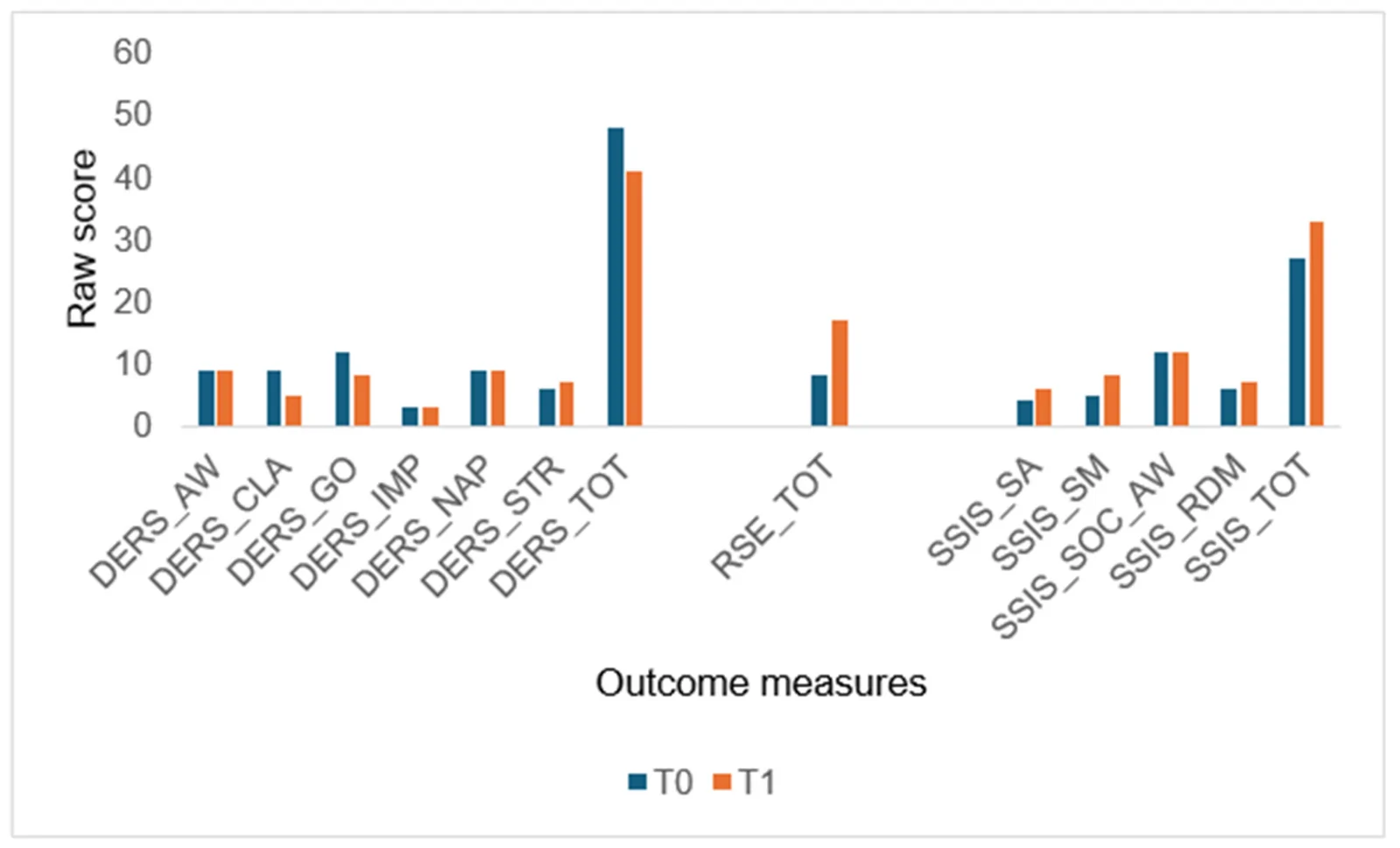
Pre- and post-intervention profile of Participant 3. Legend: DERS-SF = Difficulties in Emotion Regulation Scale; AW = awareness; CLA = clarity; GO = goals; IMP = impulse; NAP = non-acceptance; STR = strategies; TOT = total score. RSE_TOT = Rosenberg Self-Esteem Scale. SSIS-SEL = Social Skills Improvement System—Social Emotional Learning Edition; SA = self-awareness; SM = self-management; SOC_AW = social awareness; RDM = responsible decision-making.

Quantitative findings reflected moderate but clinically meaningful improvements. Total DERS-SF scores decreased, indicating reduced emotional dysregulation, with the most notable changes observed in emotional clarity and goal-directed behavior. Impulsivity, emotional awareness, and non-acceptance remained relatively stable (see Figure 3).

Socio-emotional functioning showed positive changes. Improvements were particularly evident in self-awareness and self-regulation, whereas social awareness remained stable and responsible decision-making increased slightly (see Figure 3).

A marked improvement was also observed in self-esteem, as measured by the RSE_TOT scores, suggesting the development of a more positive self-image and greater self-confidence following the intervention (see Figure 3).

#### 3.1.4. Participant 4

A.C. was an 11-year-old adolescent diagnosed with ADHD with a predominantly inattentive presentation. Generally adequate cognitive functioning was observed, alongside difficulties in executive attention, inhibitory control, and self-regulation. Clinically, she appeared emotionally reactive and showed difficulties in modulating impulsive responses, particularly in situations requiring sustained cognitive engagement or emotional processing.

During the initial stages of the intervention, A.C. demonstrated limited spontaneous participation and appeared uncomfortable in situations involving personal exposure within the group. Her engagement in reflective activities was often inconsistent, and her responses tended to remain concrete and only partially connected to the emotional themes proposed during the sessions.

Throughout the intervention, her narratives frequently focused on fantasy, conflicting, or aggressive themes, suggesting difficulties in symbolic elaboration and emotional integration. Introspective abilities remained limited across sessions, particularly during activities requiring reflection on internal emotional states and personal experiences. Nevertheless, the group progressively appeared to become a meaningful relational context for her. Over time, A.C. showed increased interest in peer interactions, greater proximity-seeking behaviors, and more active involvement during shared activities, although reflective depth and emotional elaboration remained relatively immature by the end of the intervention.

Quantitative findings were consistent with these observations. Standardized measures did not reveal clinically significant changes in emotional dysregulation or socio-emotional functioning from pre- to post-intervention. However, a slight increase emerged in self-esteem scores, suggesting modest improvements in self-perception and relational engagement within the group context.

**Figure 4:**
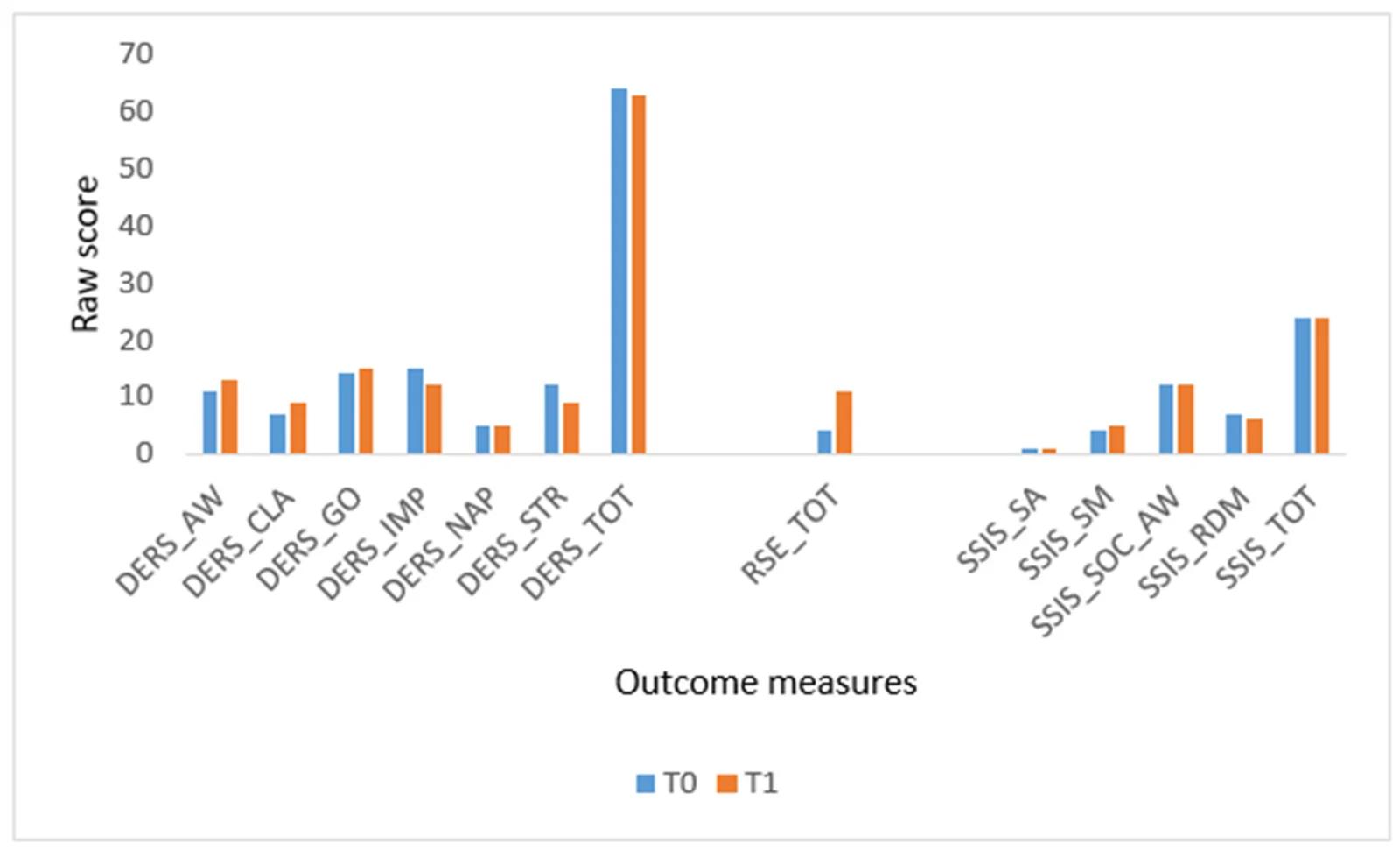
Pre- and post-intervention profile of Participant 4. Legend: DERS-SF = Difficulties in Emotion Regulation Scale; AW = awareness; CLA = clarity; GO = goals; IMP = impulse; NAP = non-acceptance; STR = strategies; TOT = total score. RSE_TOT = Rosenberg Self-Esteem Scale. SSIS-SEL = Social Skills Improvement System—Social Emotional Learning Edition; SA = self-awareness; SM = self-management; SOC_AW = social awareness; RDM = responsible decision-making.

Overall, A.C.’s clinical profile remained characterized by persistent difficulties in emotional regulation, reflective functioning, and the integration of emotional experiences. Nonetheless, the intervention appeared to facilitate greater interpersonal involvement and more positive participation in peer interactions over time (Figure 4).

### 3.2. Group-Level Quantitative Findings

Linear mixed-effects models (Equation (1)) were used to estimate pre–post changes in the assessed outcomes. The complete model estimates, including regression coefficients (β), standard errors (SE), 95% confidence intervals (CI), degrees of freedom (df), t-statistics, *p*-values, and standardized effect sizes (Cohen’s dz), are reported in Table 2.

**Figure 5:**
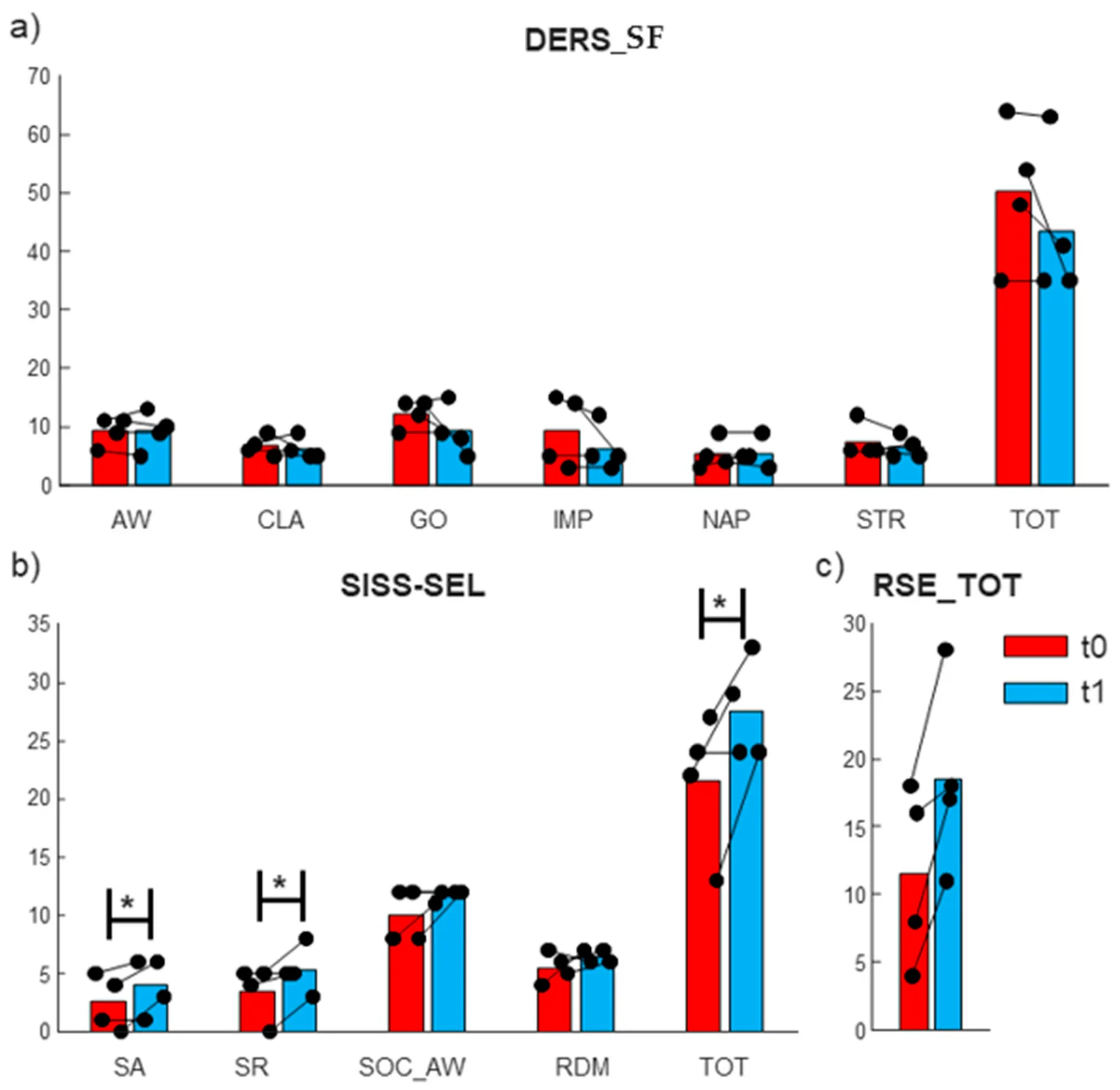
Scores on the DERS-SF (**a**), SSIS-SEL (**b**), and RSE_TOT (**c**) scales. Bars represent mean values at t0 (red) and t1 (blue). Black dots represent individual participant values, with lines connecting repeated measures. Asterisks (*) indicate statistically significant differences between t0 and t1.

Regarding emotional regulation, all DERS-SF subscales (Figure 5a) showed negative changes from t0 to t1, except for awareness and non-acceptance scores, which remained substantially unchanged (Table 2). The largest reduction was observed for the DERS-SF total score (β = −6.75, 95% CI [−16.00, 2.50], Cohen’s dz = −0.77), although the exploratory model did not provide statistical evidence of change (*p* = 0.12). Similarly, moderate reductions were observed for the goals and impulse control subscales (DERS-SF_GO: β = −3.00, Cohen’s dz = −0.66; DERS_IMP: β = −3.00, Cohen’s dz = −0.71).

In the socio-emotional domain, SSIS-SEL scores (Figure 5b) showed positive changes following the intervention. Significant increases were observed for self-awareness (β = 1.50, 95% CI [0.13, 2.87], *p* = 0.04, Cohen’s dz = 1.16), self-regulation (β = 1.75, 95% CI [0.16, 3.34], *p* = 0.03, Cohen’s dz = 1.17), and total SSIS-SEL score (β = 6.00, 95% CI [1.18, 10.82], *p* = 0.02, Cohen’s dz = 1.32). Positive changes were also observed for social awareness and responsible decision-making, although with wider confidence intervals and without statistical evidence of change (social awareness: β = 1.75, Cohen’s dz = 0.85; responsible decision-making: β = 1.00, Cohen’s dz = 0.61).

Self-esteem (Figure 5c) showed the largest standardized change among all outcomes. The Rosenberg Self-Esteem Scale score increased from t0 to t1 (β = 7.00, 95% CI [3.23, 10.77], *p* = 0.004, Cohen’s dz = 1.97), indicating a large pre–post effect.

Given the exploratory nature of this case series and the limited sample size, these findings should be interpreted primarily in terms of the magnitude and direction of change rather than statistical significance alone.

## 4. Discussion

This study describes a therapeutic writing-based intervention carried out with a group of girls with ADHD. The aim was to assess the impact on self-esteem, emotional regulation and socio-emotional skills. The results, in line with the theoretical framework underpinning the intervention, showed preliminary pre–post changes in self-esteem and in selected domains of socio-emotional skills, with the largest effects observed in self-esteem and socio-emotional competencies. No statistically supported changes were observed in emotional regulation.

These preliminary findings suggest that the intervention may be associated with processes reorganizing self-representation, shifting from a deficit-oriented perspective towards a more balanced, resource-oriented self-concept. This finding may be particularly relevant for young people with ADHD, who are often exposed to negative feedback, difficulties with peers and stigmatization—factors that can contribute to the consolidation of maladaptive beliefs about themselves and a decline in self-esteem over time [4,38].

From a cognitive perspective, these findings may be interpreted in light of the role of cognitive distortions in shaping self-perception [43]. Adolescents with ADHD are particularly vulnerable to developing biased cognitive patterns, such as the overgeneralization of failures, negative self-labeling, and selective attention to difficulties, which may consolidate into stable negative self-schemas [7,44,45].

Within this framework, therapeutic writing may contribute to processes resembling cognitive restructuring, allowing individuals to revisit personal experiences, generate alternative interpretations, and integrate more adaptive and flexible meanings [46]. Unlike traditional cognitive interventions, this process is mediated through narrative construction and symbolic elaboration, which may be more accessible and engaging for adolescents [31,32].

In the present study, the construction of fictional characters and shared storytelling may have facilitated the processes of externalization and perspective-taking, allowing participants to distance themselves from rigid self-definitions and explore alternative representations of their abilities and behaviors. Although these mechanisms were not directly assessed, they may provide a theoretical framework for interpreting the observed changes in self-perception, potentially contributing to reduced maladaptive self-labeling and a more flexible self-concept [47].

Interestingly, positive changes in socio-emotional competencies were observed, with statistically significant pre–post differences emerging particularly in self-awareness and self-regulation, while other domains did not reach statistical significance. Emotional and socio-emotional functioning represents an important target of psychosocial interventions in ADHD, with interventions addressing emotional competencies showing potential benefits in areas such as emotional awareness, self-management, self-control, and interpersonal functioning [48]. Within this framework, the changes observed in the present case series may be considered consistent with the relevant therapeutic targets for ADHD.

The group context may have provided opportunities for shared experience, interpersonal validation, and peer support. These processes could theoretically contribute to normalization, reduced perceived isolation, and the development of socio-emotional competencies; however, these potential mechanisms were not directly assessed in the present study. This interpretation is coherent with neuro-affirming perspectives emphasizing the importance of relational and contextual environments that support understanding, flexibility, and positive identity development in neurodivergent individuals, rather than focusing exclusively on symptom normalization [28].

These findings are consistent with previous literature suggesting that group-based interventions may promote social functioning and emotional competencies through processes of peer support, shared experience, and interpersonal validation [49]. In contrast, changes in emotional regulation showed greater variability and appeared to be partially influenced by baseline levels. While participants with higher initial levels of dysregulation showed more pronounced improvements, others remained relatively stable over time. This variability may reflect the complexity of the processes involved. Emotional regulation involves multiple components, including physiological, cognitive, and behavioral mechanisms, which may require more prolonged and targeted interventions to produce substantial changes [50]. However, the more consistent pre–post changes observed in self-esteem, compared with the heterogeneous changes in emotional regulation, may suggest that therapeutic writing is more closely associated with changes in self-perception and cognitive–affective representations than with emotional regulation [42,51].

From a developmental perspective, these findings may also be interpreted in light of the specific psychosocial needs of adolescence, a phase characterized by the interplay between increasing autonomy and the need for peer affiliation [52]. In this context, peer relationships represent a primary setting for identity-related processes, including self-evaluation and social comparison [53]. Accordingly, embedding therapeutic writing interventions within a group context may enhance their relevance and ecological validity, as it allows narrative reflection to occur within a shared interpersonal environment that closely reflects adolescents’ developmental context.

In particular, the integration of therapeutic writing and group processes may involve distinct but potentially complementary mechanisms. At an intrapersonal level, writing activities may facilitate internal reflection, the reinterpretation of experiences, and the restructuring of self-representations [42]. At an interpersonal level, the group context may simultaneously provide opportunities for interpersonal feedback [54], shared narratives [55], and reciprocal recognition [56,57].

These processes may interact, as self-representations developed through individual narrative elaboration may be further reinforced through sharing and validation within the group. Therefore, for adolescents with ADHD, who often report difficulties in peer relationships and negative social experiences [58], an integrated approach of this type may be particularly useful for supporting both self-perception and socio-emotional functioning.

These findings, although preliminary, provide initial evidence of pre–post changes in specific domains, particularly self-esteem. However, given the small sample size, the absence of a control group, and the exploratory nature of the analyses, they should not be interpreted as evidence of intervention efficacy. Acceptability, tolerability, and potential adverse or distressing reactions to the writing tasks were not systematically assessed, and the exclusive use of self-report measures may have introduced response bias. The limitations of this study include the small sample size and the experimental design, which limit the precision of the estimated effects and preclude the generalizability of the results. Furthermore, the absence of a control group prevents drawing causal conclusions. Finally, it is not possible to assess the stability of the changes over time, since the evaluation was conducted only at the end of the intervention. Future research should replicate the intervention with larger, controlled samples.

## 5. Conclusions

This study provides preliminary exploratory evidence that therapeutic writing in a group setting may be associated with improvements in self-perception, self-esteem, and socio-emotional functioning in adolescents with ADHD. By facilitating the reorganization of self-narratives and addressing maladaptive cognitive patterns, such interventions may represent a valuable complementary approach to support identity development and interpersonal functioning. However, these findings require confirmation through larger controlled studies.

## Figures and Tables

**Table 1:** Demographic and clinical characteristics of participants.

Participant	Age (Years)	Sex	IQ	ADHD Presentation	Comorbidities	Previous Psychological Intervention
P1	14	Female	101	Predominantly inattentive	None	Cognitive–behavioral therapy
P2	14	Female	103	Predominantly inattentive	None	Cognitive–behavioral therapy
P3	13	Female	98	Combined presentation	None	Cognitive–behavioral therapy
P4	11	Female	100	Predominantly inattentive	None	Cognitive–behavioral therapy

**Table 2:** Sample characteristics.

	β	SE	CI	df	t Statistic	*p*-Value	Cohen’s dz
DERS-SF_AW	0.00	0.61	[−1.50 1.50]	6	0.00	1.00	0.00
DERS-SF_CLA	−0.50	1.09	[−3.17 2.17]	6	−0.46	0.66	−0.20
DERS-SF_GO	−3.00	1.97	[−7.82 1.81]	6	−1.52	0.18	−0.66
DERS-SF_IMP	−3.00	1.84	[−7.49 1.49]	6	−1.63	0.15	−0.71
DERS-SF_NAP	0.25	0.54	[−1.08 1.58]	6	0.46	0.66	0.20
DERS-SF_STR	−1.00	0.71	[−2.73 0.73]	6	−1.41	0.21	−0.61
DERS-SF_TOT	−6.75	3.78	[−16.00 2.50]	6	−1.78	0.12	−0.77
SISS_SA	1.50	0.56	[0.13 2.87]	6	2.68	0.04 *	1.16
SISS_SM	1.75	0.65	[0.16 3.34]	6	2.69	0.03 *	1.17
SISS_SOC_AW	1.75	0.89	[−0.43 3.93]	6	1.96	0.10	0.85
SISS_RDM	1.00	0.61	[−0.50 2.50]	6	1.63	0.15	0.61
SISS_TOT	6.00	1.97	[1.18 10.81]	6	3.05	0.02 *	1.32
RSE_TOT	7.00	1.54	[3.23 10.77]	6	4.54	<0.01 *	1.97

The symbol “*” indicates statistical significance (*p* < 0.05).

## Data Availability

The data used and analyzed during the current study are available from the corresponding author upon reasonable request due to privacy and ethical restrictions, as they contain information that could potentially compromise participant confidentiality.
